# Chondrosternal Arthritis in Infant: An Unusual Entity

**DOI:** 10.1155/2014/652539

**Published:** 2014-10-19

**Authors:** Athina Nikolarakou, Dana Dumitriu, Pierre-Louis Docquier

**Affiliations:** ^1^Cliniques Universitaires Saint-Luc, Service de Pédiatrie, Avenue Hippocrate 10, 1200 Brussels, Belgium; ^2^Cliniques Universitaires Saint-Luc, Service de Radiologie et d'Imagerie Médicale, Avenue Hippocrate 10, 1200 Brussels, Belgium; ^3^Computer Assisted Robotic Surgery (CARS), Institut de Recherche Expérimentale et Clinique (IREC), Université Catholique de Louvain, Tour Pasteur +4, Avenue Mounier 53, 1200 Brussels, Belgium; ^4^Cliniques Universitaires Saint-Luc, Service de Chirurgie Orthopédique et Traumatologique, Avenue Hippocrate 10, 1200 Brussels, Belgium

## Abstract

Primary arthritis of chondrosternal joint is very rare and occurs in infants less than 18 months of age. Presentation is most often subacute but may be acute. Child presents with a parasternal mass with history of fever and/or local signs of infection. Clinical symptoms vary from a painless noninflammatory to a painful mass with local tenderness and swelling, while fever may be absent. Laboratory data show low or marginally raised levels of white blood cells and C-reactive protein, reflecting, respectively, the subacute or acute character of the infection. It is a self-limiting affection due to the adequate immune response of the patient. Evolution is generally good without antibiotherapy with a progressive spontaneous healing. A wait-and-see approach with close follow-up in the first weeks is the best therapeutic option.

## 1. Introduction

Primary arthritis of chondrosternal joint in infants should always be considered in the differential diagnosis when a child presents with a parasternal mass with history of fever and/or local signs of infection. As it is a very rare infection, adequate diagnosis is often delayed. The differential diagnosis includes systemic arthritis, osteomyelitis, Tietze syndrome, and SAPHO syndrome [1, 2]. Clinical symptoms may vary from a painless noninflammatory to a painful mass with local tenderness and swelling. Fever may be absent [3]. Low or high levels of white blood cells (WBC) and C-reactive protein (CRP) may be present, reflecting, respectively, the subacute or acute character of the infection.

## 2. Case Presentation

### 2.1. Case 1

A 12-month-old girl was admitted to another hospital for a parasternal mass and a three-day fever two weeks before her admission. Her medical history was clear and she had no chronic disease. At the time of admission the examination showed a swelling of the third chondrosternal joint, which was neither tender nor painful. Significant laboratory findings showed CRP 1.8 mg/dL (normal: <1 mg/dL) and WBC 10500/mm^3^. Chest radiograph was normal and ultrasound showed hyperplasic lymphatic nodes and an inflammatory mass of the third chondrosternal joint. The girl was transferred to our outpatient clinic the day after. The clinical examination revealed the presence of a right parasternal mass with no signs of local inflammation. The patient was afebrile and in excellent condition. A new ultrasound of the chondrosternal joint showed a hypoechogenic mass of the third chondrosternal joint. The girl was treated with oral oxacillin 100 mg/kg/day for 6 weeks. A total regression of the mass was noticed after 2 months. No recurrence was observed after a follow-up of 2 years.

### 2.2. Case 2

A 12-month-old boy was admitted to another institution for a parasternal mass with persistence of fever for 3 days. A chest radiograph, a thoracic CT-scan, and an ultrasound were performed without evidence concerning the nature of the mass. Laboratory findings showed a CRP 3.5 mg/dL (normal <1 mg/dL) and WBC 8.800/mm^3^ and the boy was discharged with no additional treatment. The boy was admitted to our clinic 15 days later, because of increasing pain and swelling of the mass. Medical history was significant for neonatal spontaneous pneumothorax and neonatal infection. At the time of admission, he was afebrile and in good general condition. The examination revealed a tender and painful parasternal mass. An ultrasound confirmed the presence of an abscess of the chondrosternal joint. We proceeded to a surgical drainage of the abscess and of the chondrosternal joint. Laboratory analysis showed CRP 7.9 mg/dL and WBC 15600/mm^3^. Culture of the fluid remained negative. The patient received intravenous cefotaxime and oxacillin for four days and was discharged with an additional 8-week course of oral amoxicillin-clavulanic acid treatment. After two months of follow-up, the patient was free of symptoms and no recurrence was observed after a 2-year follow-up.

### 2.3. Case 3

An 8-month-old girl was admitted in the emergency room of another institution for a parasternal mass appeared 5 days before. She had presented fever to 38.3°C only once. Clinical examination showed a very painful parasternal mass. Laboratory data showed CRP 0.5 mg/dL (normal <1 mg/dL) and WBC 9900/mm^3^. An ultrasound showed chondrosternal inflammatory mass. A percutaneous puncture of the joint liquid was performed but culture remained negative. The child was hospitalized for two days with no treatment and was transferred to our clinic after two days. At the admission she was still presenting a 2 × 2 cm parasternal mass, but the pain had disappeared and there was no more tenderness. She was discharged with oral oxacilline for 7 days. The girl had no symptoms and no relapses after two months of follow-up and presented no recurrence after a 2-year follow-up.

Three similar cases of chondrosternal arthritis in infants are reported (infants aged from 8 to 12 months). Two patients were febrile the day of admission while the third patient had been febrile 15 days before. All patients presented a parasternal mass for more than 3 days prior to consultation (Table 1). The three patients had a parasternal mass but two were painful and tender and the other one was painless. WBC count was slightly elevated in only one case and CRP was abnormal in only two patients.

**Figure 1:**
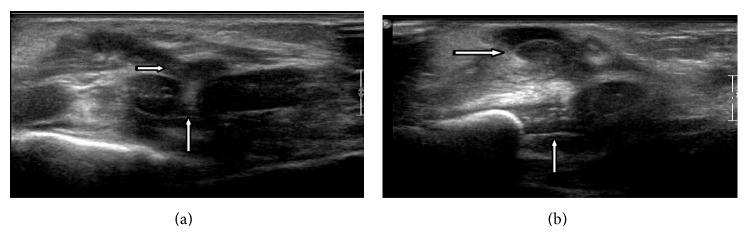
Ultrasonography of a 12-month-old boy presenting chondrosternal arthritis. (a) The route of the joint effusion (stripped arrow), into the chondrosternal joint (arrow). (b) Subcutaneous abscess (stripped arrow) from the chondrosternal joint (arrow).

Chest X-rays were normal in all patients and thoracic CT-scan performed in one patient showed no signs of osteomyelitis. Ultrasonography confirmed arthritis of the chondrosternal joint in all patients, demonstrating a hypoechogenic heterogeneous mass in relation to the chondrosternal joint (Figure 1). Blood and joint cultures were negative in all cases. Two infants received oral oxacillin and one received intravenous oxacillin and cefotaxime for three days and was discharged with an additional oral amoxicillin-clavulanic acid treatment. Total duration of the therapy ranged from 7 days to 8 weeks. Two-month follow-up showed clinical healing and no relapses were observed after a follow-up of 2 years.

## 3. Discussion

In our three cases, ultrasound was performed for the early diagnosis and for the follow-up of the chondrosternal arthritis. It is a rapid, noninvasive, nonionising and accurately identifies soft tissue collections, periosteal reaction, joint effusion, and synovial thickening [4]. Conversely, in sternal arthritis, plain chest radiograph does not show evidence of bone abnormalities. Therefore it may be avoided in case of suggestive ultrasonography. CT-scan has a greater sensitivity in precising the extension of the infection, especially retrosternal or mediastinal abscess and bone irregularities, but is performed under general anaesthesia in infants and should be considered only if ultrasound evaluation does not provide sufficient information for accurate diagnosis [5, 6].

The most common microorganism found in osteoarticular infections is* Staphylococcus aureus*, while* Streptococcus pneumoniae* and* Kingella kingae* are also found in infants especially between the ages of 2 months and 5 years [3, 7, 8]. Therefore, antibiotics against* Staphylococcus aureus* should be the empiric treatment of first choice [9]. Aspiration of the joint fluid could be performed and if the pathogen is identified, antibiotherapy is adjusted, though cultures may be negative due to the paucity of the fluid. Duration of treatment in septic arthritis varies from 3 to 6 weeks, according to several reports. Initial intravenous treatment is generally recommended and the switch to oral treatment is considered when clinical and biological features of the disease improve [5, 10]. Oral administration of antibiotics may also be proposed as a first-line treatment, as in our cases, if the arthritis presents subacute features and the patient can be regularly evaluated. Peltola et al. in a prospective randomized study suggested a 10-day antibiotic therapy in septic arthritis (initially administered intravenously). They also recommended only 1 joint aspiration for treatment of most cases of childhood septic arthritis, regardless of the infecting pathogen or anatomical site, if the clinical response is good [11].

In our three cases, treatment was different as this entity is very rare and no guidelines exist concerning the treatment. The second case seemed to be more acute and we performed a surgical drainage and a long-term antibiotherapy. Probably this invasive and aggressive treatment was exaggerated and a single needle aspiration was as efficient as well as shorter course of antibiotics.

By comparing our three cases with similar cases of the literature, it seems that chondrosternal arthritis is a self-limiting subacute affection and differs from classical acute arthritis. Description of a new entity was given by Te Winkel et al. [12]. They reported 14 cases of children (aged 7 to 50 months) with a rapidly growing sternal mass. They attributed the mass to an aseptic inflammation that they named self-limiting sternal tumor of childhood (SELSTOC). They advocated only a wait-and-see approach with close follow-up in the first weeks. In their patients, symptoms were local pain (*n* = 7 out of the 14) and/or raised body temperature (*n* = 5). For these authors, etiology of these benign aseptic inflammatory processes in the sternal region in young children remains uncertain. Roukema et al. reported 3 cases aged 11–14 months, who were seen because of sternal swelling [13]. The mass was painful. There was no history of fever. Two patients were surgically drained and the third was aspirated, and pus was drained in all cases. Culture remained sterile in all cases. Good evolution was observed in the three cases. Howard et al. reported 4 cases aged 9–16 months, who presented to the emergency room with an acute tender swelling of the sternum or sternocostal cartilage [2]. Only one case was febrile at the time of admission. One was biopsied, two others aspirated with a needle, and the fourth was observed (in the light of the experience in the previous three cases). The three first cases received a short course of antibiotics. All cases resolved within a few weeks of presentation.

In conclusion, our cases are similar to the ones reported by Te Winkel et al. [12], by Roukema et al. [13], and by Howard et al. [2]. Fever is sometimes present. Infection parameters (C-reactive protein and WBC count) may be normal or marginally raised. Echography reveals a soft-tissue swelling into the chondrosternal joint with or without abscess. Culture remains sterile in case of aspiration or surgical drainage. Antibiotherapy seems to be unnecessary.

We hypothesized a subacute infection as the etiology of this affection. No treatment is necessary as it is a self-limiting affection probably due to an adequate immune response of the patient. Evolution shows progressive healing. A wait-and-see approach with close follow-up in the first weeks is the best therapeutic option.

## Figures and Tables

**Table 1:** Clinical data of the 3 patients.

Patient's data	Case 1	Case 2	Case 3
Sex	F	M	F
Age (months)	12	12	8
Predisposing factors	—	—	—
Symptoms duration before admission	3 days	15 days	7 days
Symptoms			
Fever (>38°C)	3 days (two weeks before first admission)	Days 0–3	Day 0, once
Palpable mass	Yes	Yes	Yes
Pain	No	Yes	Yes
Biological data			
CRP (normal <1 mg/dL)	1.8	3.5	0.8
WBC	10500	8800	9900
Imaging			
Chest radiograph	Normal	Normal	Normal
Ultrasound	Inflammatory mass of the 3rd chondrosternal joint	Inflammatory mass of a chondrosternal joint	Inflammatory mass of a chondrosternal joint
Thoracic CT	Not performed	Negative	Not performed
Cultures			
Blood culture	Negative	Negative	Negative
Joint puncture	Not performed	Negative	Negative
Treatment	Oral oxacillin 100 mg/kg/day	Initial IV cefotaxime-oxacillin followed by oral amoxicilline-clavulanic acid	Oral oxacillin 100 mg/kg/day
Total duration of antibiotherapy	6 weeks	8 weeks	7 days
Follow-up	2 years	2 years	2 years
	No relapse	No relapse	No relapse

F = female, M = male, IV = intravenous, CT = computerized tomography.
